# Endovascular Treatment of Pelvic Venous Congestion Syndrome in Nulliparous Patients—Preliminary Results of 10 Years of Experience

**DOI:** 10.1007/s00270-024-03731-y

**Published:** 2024-04-30

**Authors:** Maciej Szmygin, Krzysztof Pyra, Viktor Bèrczi, Sławomir Woźniak, Łukasz Światłowski, Tomasz Paszkowski

**Affiliations:** 1 Department of Interventional Radiology and Neuroradiology, Medical University of Lublin, Jaczewskiego 8 Str., 20-954 Lublin, Poland; 2 Department of Human Anatomy, Medical University of Lublin, Lublin, Poland; 3 Department of Radiology, Medical Imaging Clinic, Semmelweis University Budapest, Budapest, Hungary; 4 Department of Gynecology, Medical University of Lublin, Lublin, Poland

**Keywords:** Pelvic venous congestion syndrome, Embolization, Nulliparity, Endovascular treatment

## Abstract

**Purpose:**

The aim of this article is to present our experience with minimally-invasive treatment for nulliparous patients with pelvic venous congestion syndrome (PVCS) with special attention to anatomical considerations, procedural and clinical outcome.

**Materials and Methods:**

In this retrospective, monocentric study, 21 patients with PVCS treated from January 2014 to June 2023 were included. The preprocedural imaging evaluation of PVCS was based on color Doppler ultrasound, contrast-enhanced CT and/or MRI. In all cases insufficient ovarian veins and/or internal iliac branches were occluded with coils and sclerosant. Procedural and clinical outcomes were measured 30 and 90 days after the procedure.

**Results:**

Average duration of pelvic pain was 44.8 ± 54.2 months (from 6 to 200) with the mean VAS-recorded pain intensity of 8.5 ± 1.1 (range from 7 to 10 where 0 was “no pain” and 10 “worst pain possible”). Most common symptoms included dysmenorrhea, dyspareunia and dysuria. Complete embolization was observed in in all cases. Targeted vessels included left ovarian vein (13/21, 62%), both ovarian veins (7/21, 33%) and left pudendal with left ovarian (1/21, 5%). Residual PVCS was noted in 1 patient. Mean VAS at 90-days after the procedure was 2.4 ± 1.4 (range from 0 to 6, *p* < 0.001). Nineteen patients (90%) were satisfied with the clinical outcome (13 “very satisfied”, 6 “satisfied”) and reported improvement in quality of life. Two patients (9.5%) reported to be “neutral” as the VAS reduction was less than 50%.

**Conclusion:**

Our study confirms that endovascular coil embolization is safe and effective in treatment of nulliparous patients with PVCS that provides very high rate of clinical success and overall satisfaction.

**Graphical Abstract:**

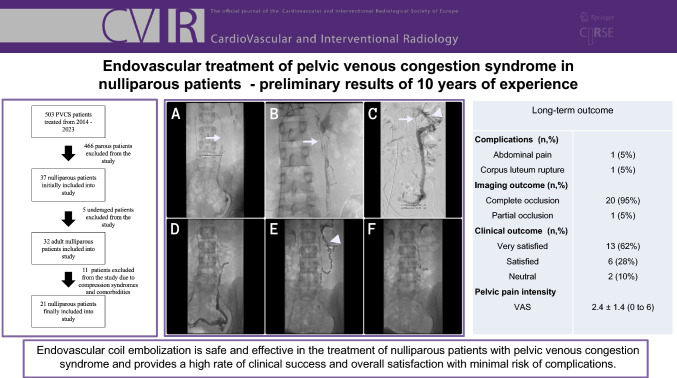

## Introduction

Chronic pelvic pain (CPP), defined as pelvic pain of more than six months duration with no evidence of inflammatory disease, affects over 10 million women worldwide and accounts for up to 20% of all gynecological appointments [1, 2]. Pelvic venous disorder (PeVD) is an umbrella term that encompasses a wide range of venous disorders, including nutcracker syndrome, May-Thurner syndrome, and pelvic venous congestion syndrome (PVCS), which may result in CPP [3]. PVCS is characterized by the dilation and dysfunction of the ovarian veins or intrapelvic venous plexuses, resulting in slow flow and reflux [4]. Although it can occur at any time in a woman’s life, PVCS is diagnosed most frequently in multiparous women as a result of increased vascular volume and vessel dilatation during pregnancy [5]. Other risk factors, including genetic predispositions, anatomical abnormalities, and hormonal factors, are hypothesized to take part in the pathophysiology of PVCS in nulliparous patients [6].

This article aimed to present a decade of experience with minimally-invasive treatment of nulliparous patients with PVCS and to review the currently available literature.

## Materials and Methods

### Study Participants

This retrospective, single-center study evaluated the outcome of endovascular treatment of nulliparous patients with PVCS from 2014 to 2023. The study was approved by the local institutional review board and was conducted in compliance with the Declaration of Helsinki. Written informed consent for the procedure was acquired from each patient. Inclusion criteria were (a) Age ≥ 18 years, (b) Diagnosis of PVCS based on clinical history and imaging examinations, including transvaginal ultrasound (TVUS), computed tomography angiography (CTA), magnetic resonance angiography (MRA), and/or digital subtraction angiography (DSA), and (c) Follow-up of at least 3 months. Exclusion criteria were (a) Multiparity, (b) PVCS caused by compression syndromes (Nutcracker and/or May-Thurner syndromes), and (c) Endometriosis, adenomyosis, and/or uterine fibroids.

All patients were asked to assess their pain and quality of life (QoL) using a visual analog scale (VAS) from 0 (“no pain”) to 10 (“worst pain possible”).

### Endovascular Procedures

From femoral access, selective catheterization of the left and/or right ovarian veins and/or internal iliac veins was conducted while patients performed a Valsalva maneuver (forced expiration of air against a closed airway) with the table in standard position. Afterward, targeted vessels were obliterated using a sandwich technique with a combination of coils (Nester and MREye coils, Cook, Inc., Bloomington, IN) and 3% sclerosant (polidocanol, mixed with air and injected in a foam form using the Tessari method with a 2 ml of liquid sclerosant in one 10 ml syringe and 8 ml of air in the other 10 ml syringe [7]). Procedural outcomes, complications, and radiation doses were noted.

### Follow-up Protocol

Postoperative embolization outcomes were assessed using clinical (a structured interview 1 month after the procedure) and imaging examinations (CD-US at 3 months follow-up).

### Statistical Analysis

The results of the satisfaction surveys were compared using a student’s t-test with a *p*-value < 0.05 indicating statistical significance.

## Results

### Patient Demographics and Clinical Characteristics

**Fig. 1:**
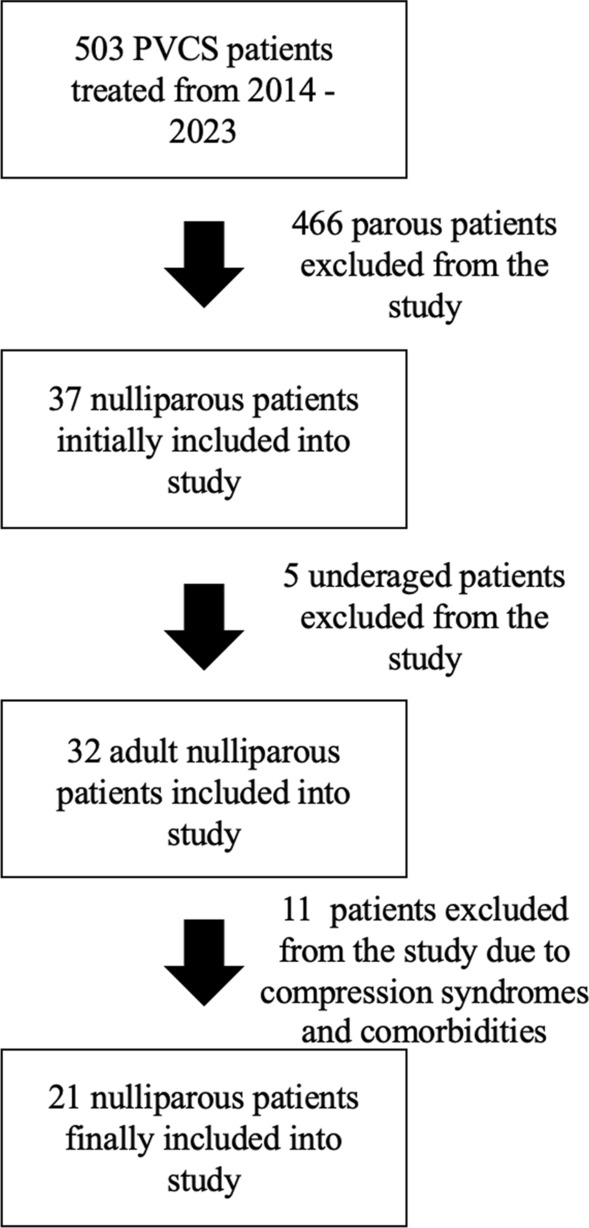
Flowchart summarizing patients’ selection process

In total, 503 patients were referred for endovascular treatment of PVCS from 2014 to 2023. From this group, 37 patients were nulliparous and were initially included. Patients who met the exclusion criteria were further excluded from the study (Fig. 1).

Twenty-one patients (mean age of 29.2 ± 7.9 years, ranging from 18 to 46) met the criteria and were included in the study. Patient demographics, duration and intensity of pain, symptoms, and oral contraception history are presented in Table 1.

**Table 1:** Demographics and clinical characteristics of the patients

*Demographic data*	
Mean age ± SD (years, min—max)	29.2 ± 7.9 (18 to 46)
*Clinical characteristics*	
Duration of pelvic pain (months, min—max)	44.8 ± 54.2 (6 to 200)
VAS (mean, min—max)	8.5 ± 1.1 (7 to 10)
*Symptoms (n, %)*	
Dyspareunia	16 (76%)
Dysmenorrhea	15 (71%)
Pain after prolonged standing	14 (67%)
Dysuria or urinary urgency	11 (52%)
Varices (legs)	7 (33%)
Vulvar varices	6 (29%)
Use of oral contraception (n,%)	8 (38%)

### Pre-Procedural Imaging Findings and Endovascular Treatment

**Fig. 2:**
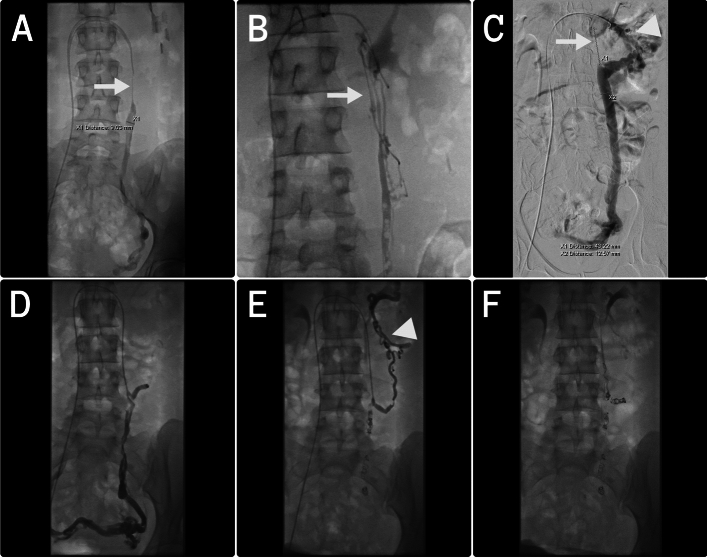
Typical angiographic findings of hypoplastic proximal part of ovarian veins (**A**, **B**, **C** -white arrows) in nulliparous patients. Additionally, visible collateral blood supply originating from distal part of left renal vein (**C**, **E** – white triangles). **D**–**F** – embolization procedure in a 24-year old patient with PVCS diagnosis and symptoms duration of 10 months. Control DSA after the procedure in discloses complete occlusion of the left ovarian vein

In the majority of cases, unilateral left ovarian vein (LOV) embolization was performed (13/21, 62%). In 7 patients (33.3%), both ovarian veins were occluded, and in 1 patient (5%), the left pudendal vein was embolized in addition to the LOV. Interestingly, 8 patients (38.1%) had hypoplasia of the proximal LOV (Fig. 2).

Endovascular treatment details, including vessel diameters, anatomical variants, radiation doses, complications, and outcomes, are shown in Table 2.

**Table 2:** Procedural details of the patients

Procedural details	
Embolized vessels (n, %)	
Left ovarian vein	31 (62%)
Both ovarian veins	7 (33%)
Left pudendal vein	1 (5%)
*Maximum vein diameter in mm (range, ±)*	
Left ovarian vein	8.14 ± 1.74 mm (6 to 13 mm)
Right ovarian vein	5.3 ± 0.93 mm (4 to 8 mm)
*Anatomical variants (n, %)*	
Left renal vein variant	1 (10%)
Hypoplastic LOV*	8 (38%)
*Number of procedures required*	
1	18 (86%)
2	3 (14%)
Mean radiation dose in mGy (± , range)**	178 mGy ± 34.8 (120 to 253)

*Proximal diameter of the vessel < 0.5 compared with distal diameter

**Calculated as a reference air kerma

### Follow-up

Overall, the follow-up time was 23.2 ± 6.2 months (5 to 64 months). During this period, one patient reported dysmenorrhea, which occurred during two menstruations following the procedures. Another patient was diagnosed with a rupture of the corpus luteum six weeks after the procedure. She did not require surgical treatment. Imaging examinations disclosed a complete or near-complete exclusion of the dilated pelvic veins in 20 cases (95.2%). Residual PVCS was noted in 1 patient (4.8%).

**Fig. 3:**
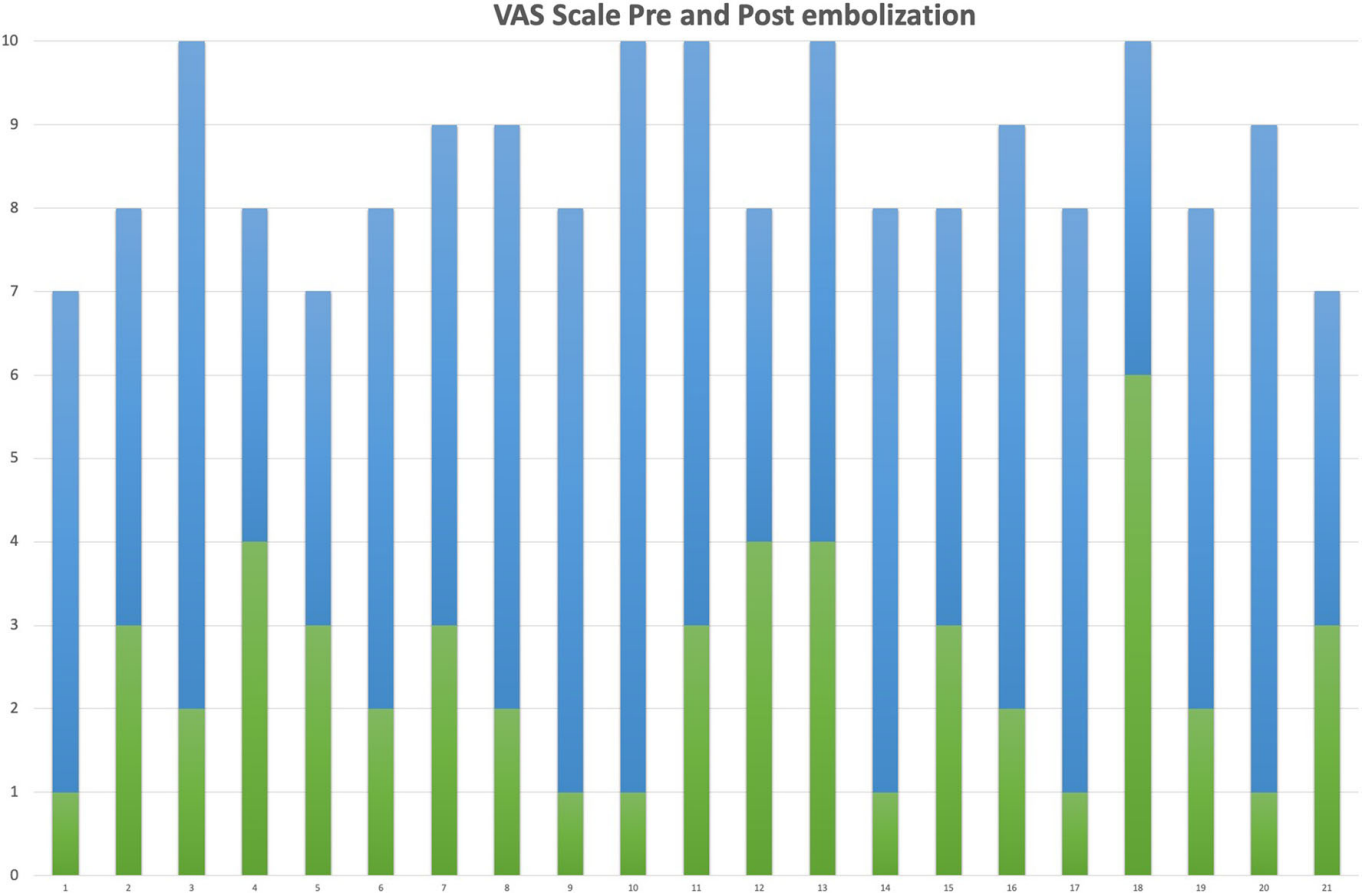
VAS scale changes in patients before (blue) and after (green) embolization

Nineteen patients (90.5%) were satisfied with the clinical outcome (13 were “very satisfied” and 6 were “satisfied”). The mean pelvic pain intensity rated by a VAS at 90-days after the procedure was 2.4 ± 1.4 (ranging from 0 to 6, Fig. 3). Two patients (9.5%) reported their outcome to be “neutral” —in both cases, repeat embolization, during which right ovarian vein occlusion and thorough pelvic venography, was performed. Despite this, the VAS pain reduction was < 50%.

The data obtained during follow-up were compared with the pre-embolization results, and a statistically significant improvement in pain evaluated using the VAS Scale was observed (*p* < 0.001). Long-term results of the endovascular treatment are presented in Table 3.

**Table 3:** Long-term outcome

Long-term outcome MEAN ± SD (mean 23.2 ± 6.2, range from 5 to 64 months)	
*Complications (n, %)*	
Abdominal pain	1 (5%)
Corpus luteum rupture	1 (5%)
*Imaging outcome (n, %)*	
Complete occlusion	20 (95%)
Partial occlusion	1 (5%)
*Clinical outcome (n, %)*	
Very satisfied	13 (62%)
Satisfied	6 (28%)
Neutral	2 (10%)
Pelvic pain intensity-VAS (mean, min—max)	2.4 ± 1.4 (0 to 6)

Three patients (14.3%) reported successful pregnancy within 5 to 64 months after treatment.

## Discussion

This study was designed to present our experience with nulliparous patients diagnosed with PVCS who underwent endovascular embolization. To our knowledge, it is the first study focusing entirely on providing detailed data in nulliparous patients—other studies included nulliparous patients, but they were not described separately in the details—see Table 4 [8–10]. In our opinion, this group deserves special attention, as PVCS is equally prevalent among parous and nulliparous women, according to some authors [11].

**Table 4:** Review of currently available literature on treatment of PVCS in nulliparous patients

Authors	N° of nulliparous patients/total patients in the study	Symptoms	Findings	Treatment	Outcome
Senechal et al. [12]	39/327 (11.9%)	N/A	High prevalence of Nutcracker syndrome	Liquid embolics—Onyx	92.3% improvement overall
Chung et al. [13]	4/52 (7.7%)	Lower back pain, abdominal pain,	N/A	Coil embolization/ hysterectomy	N/A
Kim et al. [14]	80/127 (63%)	Chronic pelvic pain	N/A	Coil embolization	Significant improvement in overall pain all symptom categories, for parous and nulliparous patients, however, no significant difference between parous and nulliparous patients
Current study	21/21 (100%)	Dysmenorrhea, dyspareunia, dysuria or urinary urgency and pain after prolonged standing	High prevalence of hypoplasia of the proximal part of the ovarian veins	Sandwich technique with coils and 3% sclerosant	Imaging—95% success Clinical—pain reduction in all cases Satisfaction—90%

In terms of procedural outcomes, we observed a complete occlusion in all patients with only 2 minor complications. Overall, the vast majority of the patients (90.5%) claimed to be satisfied after the treatment and reported significant clinical improvement. All patients were assessed using postoperative clinical and imaging examinations. The mean pelvic pain intensity significantly decreased from 8.5 ± 1 to 2.4 ± 1.4 after embolization (*p* < 0.001). These results confirm the high rate of clinical success and satisfaction described in previous studies [12, 13].

Interestingly, in our cohort, hypoplasia of the proximal part of the LOV was a common finding—38.1% of all patients. According to Szary et al., who included nulliparous patients in their cohort, 37.4% demonstrated the presence of various anatomical factors and developmental variations of the venous system, and 8.8% from this group demonstrated LOV anomalies [14]. There is no evidence of LOV hypoplasia leading to the development of PVCS currently available in the literature, our findings require validation in further studies.

A concern with any intervention involving the reproductive system in women is its potential impact on future fertility. Several studies reported no hindrance or reduction in female reproductive ability, no differences in pre-and post-embolization levels of LH or FSH, and pregnancies and live births in patients undergoing endovascular treatment due to PVCS [15–17].

Our study has several limitations. A small sample size, lack of a control arm, and lack of long-term follow-up are the most important ones.

## Conclusion

In conclusion, our study confirms that endovascular coil embolization is safe and effective in the treatment of nulliparous patients with PVCS and provides a high rate of clinical success and overall satisfaction with minimal risk of complications.
